# Successful case of olaparib treatment for castration-resistant prostate cancer with multiple DNA repair gene mutations: Use of comprehensive genome profiling for treatment-refractory cases

**DOI:** 10.1016/j.eucr.2022.102210

**Published:** 2022-08-30

**Authors:** Yukiyoshi Hirayama, Minoru Kato, Kaoru Kimura, Taiyo Otoshi, Takeshi Yamasaki, Junji Uchida

**Affiliations:** a Department of Urology, Osaka Metropolitan University Graduate School of Medicine, Osaka, Japan; b Department of Urology, Osaka City General Hospital, Osaka, Japan

**Keywords:** Castration-resistant prostate cancer, DNA repair, Poly ADP-ribose polymerase inhibitor

## Abstract

Herein, we report a case of a 59-year-old man with advanced castration-resistant prostate cancer with rectal invasion. Multimodal treatment, including drug therapy, surgery, and radiation therapy was sequentially performed; however, lymph node metastases repeatedly occurred. Tumor genomic profiling using FoundationOne CDx identified pathogenic alterations in three DNA repair genes, including BRCA2 frameshift mutation. Olaparib, a poly-ADP ribose polymerase inhibitor, showed marked response. Castration-resistant prostate cancer with multiple DNA repair genes was successfully treated with olaparib; comprehensive genome profiling can lead to its optimal clinical management.

## Highlights

• Castration-resistant prostate cancer was successfully treated with olaparib.
• The prostate cancer had pathogenic alterations in three DNA repair genes.
• Genome profiling can lead to optimal clinical management in prostate cancer.

## 1 Introduction

Although tumor genomic profiling (TGP) is becoming accessible in daily practice, optimal management strategies using next-generation sequencing (NGS) data analysis for metastatic prostate cancer (PCa) remain unclear. Herein, we report a case of metastatic castration-resistant prostate cancer (CRPC) that was successfully treated with olaparib based on TGP.

## 2 Case presentation

A 59-year-old man presented at a local hospital with complaints of frequent urination; his serum PSA level was 105 ng/ml. Prostate needle biopsy revealed PCa with a Gleason score of 5 + 5 (10). Magnetic resonance imaging (MRI) revealed PCa with rectal invasion and pelvic lymph node metastasis. No visceral or bone metastases were detected on computed tomography (CT) or bone scans. The patient was diagnosed with cT4N1M0 PCa, with no family history of PCa.

First, the patient received combined androgen blockade; PSA decreased to a nadir of 0.162 ng/ml but gradually began to rise. The patient was referred to our hospital for further treatment. Although PSA was controlled at 0.632 ng/ml, MRI revealed local tumor progression; thus, the patient was sequentially treated with docetaxel and cabazitaxel. He was then switched to enzalutamide following tumor progression and severe pedal edema; however, PSA rose to 4.19 ng/ml and MRI showed progression of rectal invasion. Serum neuron-specific enolase was within normal limits. Since there was no distant metastasis, the patient underwent total pelvic exenteration. Histopathological examination revealed adenocarcinoma with Gleason score 5 + 4 = 9, EPE1, RM0, and pT4 with rectal invasion. No lymph node metastasis was observed.

**Fig. 1:**
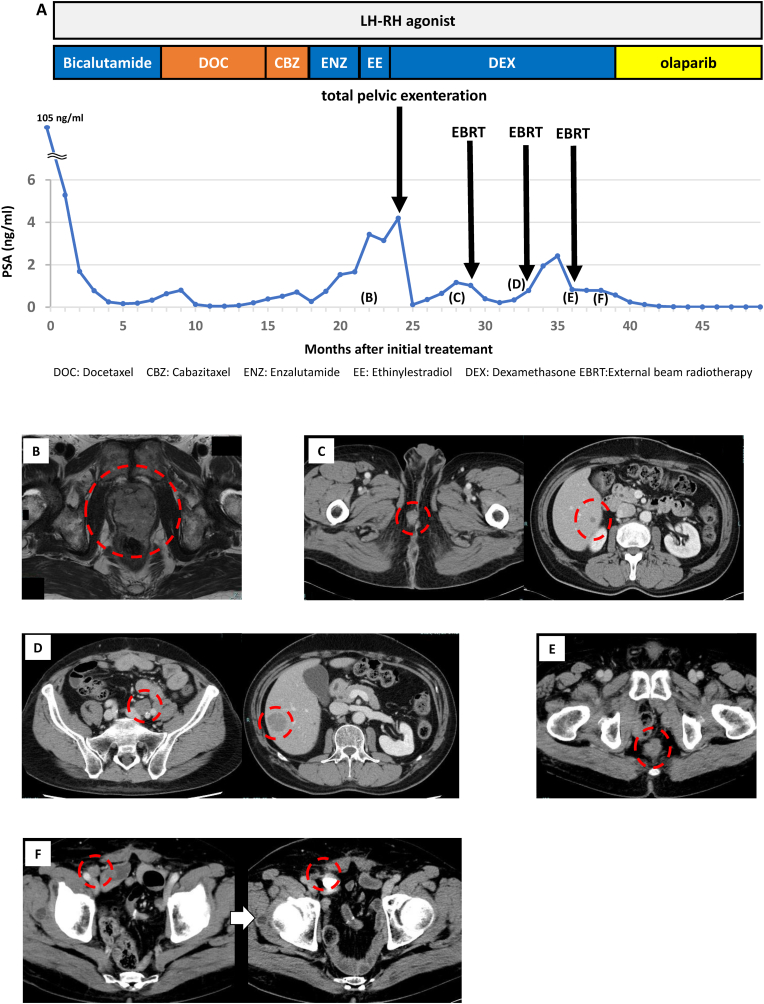
Clinical course of the case (A) Time-course change in therapies and serum PSA levels. (B) MRI image revealed prostate cancer with invasion of the rectum. (C) CT revealed local recurrence and liver metastasis. (D) CT revealed lymph node metastasis and liver metastasis. (E) CT revealed lymph node metastasis in the pelvic floor. (F) Lymph node metastasis before and after the treatment with olaparib.

Following surgery, the PSA level declined to 0.113 ng/ml but immediately began to rise again. Follow-up CT revealed local recurrence and liver metastasis; hence, stereotactic radiation therapy was performed. Subsequently, CT scans repeatedly revealed pelvic lymph node metastases and liver metastasis; the patient underwent radiotherapy for each recurrence. Although the metastases shrunk after radiotherapy, a new lymph node metastasis emerged just 1.5 months after the last treatment (Fig. 1). Therefore, the FoundationOne CDx next-generation sequencing test (F1CDx) was performed using the prostate tissue sample to identify potential treatment regimens. As shown in Table 1, AR amplification (estimated CN: 71), ATR splice variant (7504-1G>T), MYC amplification (estimated CN: 9), RAD21 amplification (estimated CN: 11), SPEN nonsense mutation (R758*), and BRCA2 frameshift mutation (I1859fs*3) were identified as clinically significant variants. Other than olaparib for BRCA2 mutation, there were no available treatments or clinical studies in the present case. Olaparib was started, and his PSA level decreased to below detectable levels, and lymph node metastasis regressed. There was no regrowth of metastases, and PSA values remained at ≤ 0.06 ng/mL 12 months after olaparib was initiated.

**Table 1:** Clinically significant gene alterations in the case.

GENE alteration	VAF (%)	eCN	status
BRCA2 I1859fs*	85.2%		Likely pathogenic
AR amplification		17.27	Pathogenic
ATR splice site 7504-1G>T	16.7%		Likely pathogenic
MYC amplification		2.2	Pathogenic
RAD 21 amplification		2.91	Pathogenic
SPEN R758*	26.3%		Likely pathogenic

VAF: Variant allele frequency eCN: estimated copy number.

## 3 Discussion

In the PROfound trial, olaparib, a poly-ADP ribose polymerase (PARP) inhibitor, demonstrated overall survival benefit in patients with metastatic CRPC with a homologous recombination repair (HRR) gene mutation.^1^ BRCA plays a critical role in HRR, which is a major pathway for DNA double-strand breaks (DSBs). DSBs can lead to cell death or genomic instability when unrepaired or incorrectly repaired. PARP is also an important protein in the DNA repair pathway; PARP inhibitors effectively kill tumor cells with defective HRR, similar to BRCA mutant cells, while sparing normal cells. This mechanism is called “synthetic lethality.”

BRCA2 mutations not only serve as markers of PARP inhibitor sensitivity but also increase the risk of aggressive PCa and are associated with a higher incidence of PCa.^2^ This patient had pathogenic alterations in two DNA repair genes; ATR and RAD21 in addition to the BRCA mutation. The aggressive disease course and the marked response to olaparib might attribute to these multiple genetic alterations. It has been reported that the number of DNA repair gene variants are associated with increased neoantigen burden.^3^ Moreover, it is possible that the prior treatment multiple courses of radio therapy affected the effect of olaparib in this case because radiotherapy also causes DNA damage. A phase I/II study of combination olaparib and radium-223 in metastatic CRPC patient is ongoing to evaluate the synthetic effect of PARP inhibition and radiotherapy (NCT03317392).

Next-generation sequencing-based comprehensive genome profiling (CGP) is fast becoming part of routine clinical practice and can provide potential treatment regimens and help guide clinical management by analyzing cancer-associated gene alterations. However, this does not mean that all patients benefit from the CGP tests. In a prospective trial investigating the utilization of NGS-based analysis in Japan, 59.4% of patients had actionable gene aberrations; however, only 13.4% of patients received therapy with a drug targeting aberration.^4^ In the SHIVA trial, off-label use of targeted therapy based on tumor molecular profiling did not improve progression-free survival compared to conventional therapy.^5^ This suggests that there is insufficient targeted treatment or clinical trials available for patients with pathogenic gene alterations; therefore, novel therapeutic approaches are urgently needed to improve accessibility to matched targeted therapies.

## 4 Conclusion

We presented a case of locally advanced CRPC with BRCA2 mutation that markedly responded to the PARP inhibitor, olaparib. CGP tests provide possible therapeutic options and have become accessible for daily practice; however, clinicians still face challenges regarding the optimal use of CGP tests in CRPC patients. We believe that the accumulation of real-world data, including case reports and clinical trials, will serve as a platform for developing future management strategies based on tumor genetic status.

## Informed consent

Written informed consent was obtained from the patient for publication of this case report.

## Declaration of competing interest

The authors declare no conflict of interest.

## List of abbreviations

CGP: comprehensive genome profiling
CRPC: castration-resistant prostate cancer
DSBs: double-strand breaks
HRR: homologous recombination repair
NGS: next-generation sequencing
PARP: poly-ADP ribose polymerase
PCa: prostate cancer
